# Critical flicker fusion frequency: confounders and caveats

**DOI:** 10.1007/s00421-025-05935-7

**Published:** 2025-08-27

**Authors:** Thomas Muth, Jochen D. Schipke, Anne-Kathrin Brebeck, Sven Dreyer

**Affiliations:** 1 Faculty of Medicine, Institute of Occupational, Social, Environmental Medicine, Heinrich-Heine-University Düsseldorf, Düsseldorf, Germany; 2 Artemis MVZ Frankfurt, Main, Frankfurt, Germany; 3 Hyperbaric Oxygen Therapy, University Hospital Düsseldorf, Düsseldorf, Germany; 4 Research Group Experimental Surgery, University Hospital Düsseldorf, Moorenstrasse 5, 40225 Düsseldorf, Germany

**Keywords:** Critical flicker fusion frequency, Divers, Age, Lighting, Nitrogen, Oxygen

## Abstract

**Introduction:**

Critical flicker fusion frequency (cFFF) measures visual perception, with higher values indicating better performance. It is widely used to assess the effects of breathing gases in diving and hyperbaric environments.

**Aim:**

Identify confounders explaining inconsistent cFFF study results.

**Methods:**

Four experiments using a manual flicker device in volunteers with healthy eyes: 1. cFFF was measured in 174 recreational divers to examine demographic effects. 2. One male diver was tested daily over 85 days. 3. In 28 divers, cFFF was measured before and after 5 min of normobaric oxygen. 4. In 24 divers, cFFF was tested with and without diving masks, plus a condition with diagonal flicker light on the mask.

**Results:**

1. Mean cFFF: 39.2 ± 5.1 Hz; decreased with age (*p* < 0.001), no gender/BMI effect. 2. Mean: 43.4 ± 2.1 Hz; stable across lighting. 3. Thresholds differed by frequency order; minimal O₂ effect. 4. Masks had no effect, but diagonal light reduced cFFF (*p* < 0.01).

**Conclusion:**

cFFF varied by 13% across divers and 5% in a single diver. Age and observer-light geometry influenced results. Oxygen had minor impact. Findings support using age-specific norms and considering perceptual confounders in diving research.

## Introduction

The critical flicker fusion frequency (cFFF)—a concept with a remarkably long history (Braunstein 1903)—defines the temporal resolution of the visual system, with higher cFFF values indicating greater perceptual accuracy. The ability to perceive flicker varies widely across species, reflecting adaptations to different ecological niches. For instance, cFFF values range from 1 to 15 Hz in various snail species, while some birds can perceive flicker at frequencies around 100 Hz (Lafitte et al. 2022).

In humans, reported cFFF values vary considerably, likely due to differences in equipment and methodological approaches. Studies have documented ranges from about 10 to 60 Hz (Hanser and Scholtyssek 2000), and from 50 to 90 Hz (Mankowska et al. 2021). For normal adults, cFFF is typically around 35 to 40 Hz (Gautam and Vinay 2020), which aligns with the temporal resolution limits of human photoreceptors in the central retina—generally below 50 Hz (Kircheis et al. 2014). Flicker fusion occurs because, above the cFFF threshold, the receptor potential can no longer distinguish individual changes. Instead, the electrical responses merge, resulting in a constant level of depolarization that the central nervous system interprets as continuous light (Muth et al. 2023).

The relationship between cFFF, cortical activation, and central nervous system (CNS) fatigue was first demonstrated by Simonson and Brožec (1952), who proposed that a decrease in cFFF indicates increased CNS fatigue (Brozek and Keys 1944). After a period of limited application, cFFF was revived as a tool for assessing CNS fatigue and cortical arousal (Schwarz et al. 1993) and has more recently been used to evaluate alertness and cognitive performance (Piispanen et al. 2021). Notably, cFFF has also been employed as one of the criteria in the selection of professional divers (Ozyigit and Egi 2014).

The effects of hyperbaric pressures of nitrogen, oxygen, and argon on cFFF were first investigated in rhesus monkeys over 50 years ago (Burns 1971). Subsequent studies extended these investigations to humans, including during a 27-day dry saturation exposure to Heliox (pO₂: 0.38–0.52 bar) at pressures up to 62 bar (Seki and Hugon 1976). In another study, a 17-day dry saturation exposure to Heliox at 18.6 bar did not affect cFFF but significantly increased peripheral visual thresholds, interpreted as evidence of severe psychological and physiological stress rather than a gas-induced effect (O’Reilly et al. 1977).

Today, cFFF is recognized as a valuable tool for examining the impact of breathing gases under increased pressures on fatigue, cortical arousal, alertness, and cognitive abilities. It has been applied in various settings, including hyperbaric chambers, breath-hold diving, scuba diving, and rebreather diving.

The following sections review key studies, highlighting the often controversial findings in this field.

### Hyperbaric chamber

In one study, the effects of 0.7, 1.4, and 2.8 bar O₂ on neuronal excitability were measured using cFFF. Results showed a dose-dependent response: 0.7 bar O₂ had no effect, while 1.4 bar O₂ significantly decreased cFFF. At 2.8 bar O₂, cFFF levels recovered, suggesting intertwined pathways of cFFF and oxygen toxicity (Kot et al. 2015). Contrasting findings have also been reported: for example, at 6 bar air, reaction times in performance tests increased, while cFFF increased slightly by 2.5 ± 2.8% (Tikkinen et al. 2013). Breathing Nitrox40 increased cFFF at 4 bar, but after 20 min, cFFF returned to pre-dive levels, indicating an early improvement in cognitive abilities and cerebral arousal due to O₂-dependent activation in the prefrontal cortex (Lafère et al. 2019).

In another study, breathing 100% O₂ at 1 and 2.8 bar significantly decreased cFFF, while it remained unchanged at 6 bar, leading the authors to question the sensitivity of cFFF as a tool for assessing gas narcosis in divers (Vrijdag et al. 2020). A subsequent commentary suggested that protocol variations may have influenced these results and cautioned against relying solely on cFFF for measuring gas narcosis (Kot and Winklewski 2021). These authors also noted that oxygen's beneficial effects may become toxic beyond a certain threshold.

### Breath-hold diving

In apneists, cFFF increased by 2% after maximal dynamic apnea, suggesting that such dives do not significantly alter CNS fatigue or cortical arousal, likely due to insufficient hypoxia duration (de Asís Fernández et al. 2019).

### SCUBA diving

Following a no-decompression dive with Nitrox32 (Nx32), 219 divers reported less fatigue compared to air dives, accompanied by a 6% decrease in cFFF with air and a 4% increase with Nx32, suggesting complex nervous system interactions influenced by the breathing gas used (Lafere et al. 2010). Similarly, Nitrox dives were associated with better preserved alertness and short-term memory, even when cFFF was not directly measured (Brebeck et al. 2017).

In a 20-min no-decompression dive to 33 m with air, cFFF increased to 104 ± 5% at depth but decreased to 94 ± 4% during bottom time, with impairments persisting 30 min after surfacing (Balestra et al. 2012). Another comprehensive study found that environmental conditions did not affect inert gas-induced narcosis, as cFFF increased by 5% at depth and decreased by 6% before surfacing, regardless of the environment (Lafère et al. 2016).

### Rebreather diving

Although closed-circuit rebreathers (CCRs) are increasingly used, data on their effects on cFFF are limited. One study showed that divers breathing air, trimix, or heliox to 50 m experienced a slight increase in alertness and arousal, suggesting that nitrogen and oxygen, alone or in combination, can affect neuronal excitability (Rocco et al. 2019). Another study found a 12% increase in cFFF during a CCR dive to 45 m in an ice-covered quarry, which returned to baseline after surfacing, indicating no clinically relevant cerebral impairment (Piispanen et al. 2021). In a further study, divers assessed cFFF before and after a bounce dive to approximately 100 m while breathing trimix, with no significant differences between pre- and post-dive values (Dugrenot et al. 2021).

These diverse findings raise questions about the effects of respiratory gases under pressure, particularly nitrogen, oxygen, and gas bubbles (Lafere et al. 2010). Some studies suggest that these gases can trigger neuronal excitability or depression in a dose-dependent manner (Rocco et al. 2019). An additional challenge is the role of carbon dioxide, which has also been described as influencing cFFF in a depth-dependent manner (Freiberger et al. 2016).

Future research should further explore underlying mechanisms, particularly the lipid theory (Wlodarczyk et al. 2006) and the gas-protein theory, which posits that inert gases exert their effects by binding to neurotransmitter protein receptors (Rostain and Balon 2006).

To provide a detailed and accurate assessment of potential confounders and caveats in cFFF measurements, we developed four distinct experiments. This approach builds on the foundational work of Erlick and Landis (1952), who identified more than ten factors that can confound "true" cFFF values (Erlick and Landis 1952), including age, colour, area, and the light–dark ratio. These and other confounders have been extensively described in a recent own review (Muth et al. 2023).

Our four experimental approaches were as follows:

*Experiment 1:* Collect statistical properties of demographic data and investigate dependencies of cFFF on age, gender, and BMI.

*Experiment 2*: Collect statistical data from one individual over 85 successive days under different lighting conditions.

*Experiment 3*: Examine the effect of a 5-min oxygen inhalation on cFFF, along with the influence of the direction of frequency change.

*Experiment 4:* Investigate the dependency of cFFF on the angle between the flicker light beam and the eyes.

Aim: By implementing these four experiments, we aim to provide an even more comprehensive understanding of the factors influencing cFFF measurements, thereby improving the reliability and comparability of future research in this area.

## Participants/methods

### Participants

A total of 174 eye-healthy, experienced divers (46 females; 26%) with an average age of 46.2 ± 14.5. (mean ± SD) years (m/f: 45.0 ± 14.5 / 49.5 ± 14.0 years) participated in experiment 1 of this study. With an average height of 177.7 ± 8.9 cm (m/f: 180.9 ± 6.7 / 169.1 ± 8.4 cm) and an average body mass of 82.0 ± 13.9 kg (m/f: 85.5 ± 12,1 / 72.2 ± 14.1 kg), the average BMI was 25.9 ± 3.4 kg/m^2^ (m/f: 26.1 ± 3.3 / 25.2 ± 3.6 kg/m^2^). Closer examination revealed that roughly 20% of the divers were classified as overweight. In experiments 3 and 4, representative subgroups of the total group were included.

The planned investigations were presented to and approved by the Ethics Committee of the Medical Faculty of the Heinrich-Heine-University, Düsseldorf under No 2023–2493 in accordance with the declaration of Helsinki (Harriss et al. 2017).

### Methods

A manually operated flicker device (Scaleo flicker, RP-Engineering GmbH, D-Esslingen) was employed in all four experiments due to its ease of use and suitability for future underwater studies. The device allowed continuous adjustment of flicker frequency via a handwheel. As the stimulus frequency approached the critical flicker fusion frequency (cFFF) threshold, participants became more attentive, resulting in slower and finer adjustments of the handwheel—typically at a rate of approximately 0.2 Hz per second—until the participant signaled detection of fusion with the predetermined "OK" response. In cases where participants were uncertain about their decision or when a clear outlier was observed, the measurement was repeated from the beginning.

Subjects: All measurements were performed by a single observer (JDS), who provided each participant with a detailed explanation of the experimental protocol and demonstrated both flickering and steady light conditions.

The light source was an LED with a color temperature of 8000 K (bluish light) if the ratio is 1:1, the light and dark phases are of equal duration. At a flicker frequency of 20 Hz, for example, both phases last 0.05 s. If the frequency increases further, the duration of each phase shortens accordingly while maintaining a constant light-to-dark ratio—an approach that has been proposed in the literature (Kelly 1961; Sekuler et al. 2002).

A digital display behind the front panel showed the individual frequency to one decimal place, which was not disclosed to participants but was recorded for analysis.

As geometric variables affect cFFF (Hosokawa et al. 1997), all experiments maintained a fixed distance of 1.5 m between the participant and examiner, with the participant looking slightly downward at the handheld device. In Experiment 4, the retinal plane and light beam purposely formed a 30° angle.

The only participant in Experiment 2 is the senior author (JDS). This participant (82) occupied the same seat in an enclosed room near a window on 85 consecutive days during the early morning hours. Under these constant conditions, the cFFF threshold was determined starting from low frequencies. For each respective cFFF threshold, the prevailing ambient lighting was categorized as artificial light, sunlight, or cloudy daylight.

In Experiment 2, oxygen was supplied from a standard cylinder, and administration was ensured using a conventional regulator.

Measurements for Experiments 1, 3, and 4 were conducted in the late afternoon, whereas those for Experiment 2 took place in the early morning hours. Experiment 1 included 174 participants, while Experiments 3 and 4 involved 28 and 24 divers, respectively, all of whom were randomly recruited from the total cohort.

### Statistics

Mean values and frequencies are presented to describe the results.

The prerequisites for the use of variance analysis methods were not available for all questions. Non-parametric methods were therefore used to examine statistical differences.

Experiment 1: The covariation of cFFF, age and BMI was analyzed with a Pearson correlation. The differences in cFFF for gender and age groups were tested using the Kruskal–Wallis test.

Experiment 2: The cFFF under different light conditions was compared using the Kruskal–Wallis test.

Experiment 3: The differences in the direction of frequency change and respiratory gases were analyzed using the Friedman test and Wilcoxon test.

Experiment 4: The same procedures were used to test the influence of the diving mask and the light beam.

The statistical program SPSS (version 29.01) was used for all calculations.

## Results

**Fig. 1:**
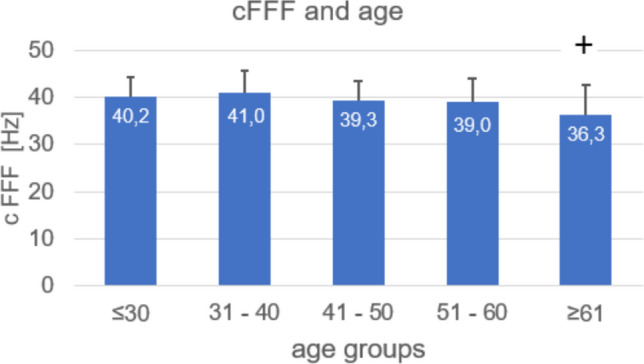
cFFF and age of 174 participants. In the 60- and the 70-year-olds, cFFFs were significantly decreased vs the younger age groups ^+^*p* < 0.001 vs all younger age groups

Experiment 1**:** Among the 174 participants, the average critical flicker fusion frequency (cFFF) was 39.2 ± 5.1 Hz (mean ± standard deviation), with males (*n* = 128) averaging 39.6 ± 4.6 Hz and females 38.2 ± 6.2 Hz (*n* = 46). No significant differences for cFFF were found for gender and no correlations between cFFF and BMI. However, cFFF showed a significant decline with age (*r* = – 27, *p* < 0.001), particularly in participants aged 60 and 70 years compared to younger groups (Fig. 1). Interestingly, the standard deviation of cFFF was significantly greater in these older age groups than in younger participants. These findings remained consistent when analyzed separately by gender.

**Fig. 2:**
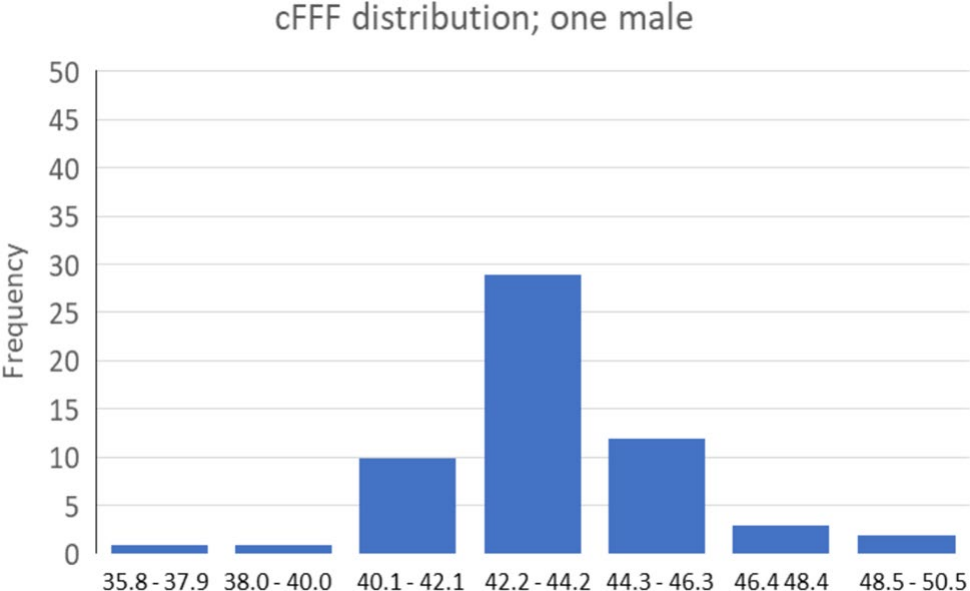
Variation of 85 cFFF values in one and the same male partcipant

Experiment 2*.* For one male participant, increasing the frequencies over 85 days, resulted in cFFFs of 43.4 ± 2.1 Hz (Fig. 2). As expected, the standard deviation in this strictly controlled setting was roughly 5%, i.e. only half of that for the total group in Experiment 1. It is pointed out that the examiner and the participant were the same person.

**Fig. 3:**
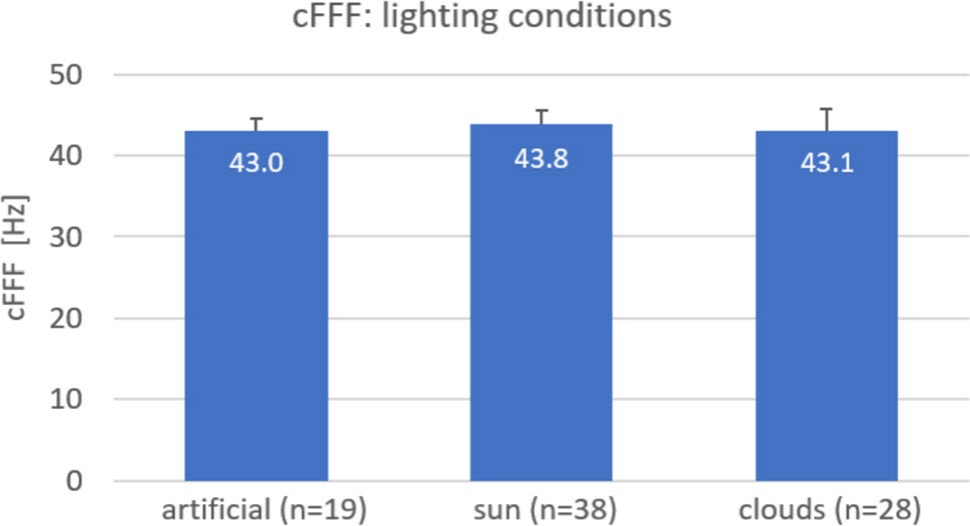
Effect of three lighting conditions over 85 subsequent days of one male participant

This experiment also showed that cFFFs did not depend on the lighting conditions (Fig. 3). An additional examination of the cFFF values revealed no time-dependent changes within the 85 days of measurements.

**Fig. 4:**
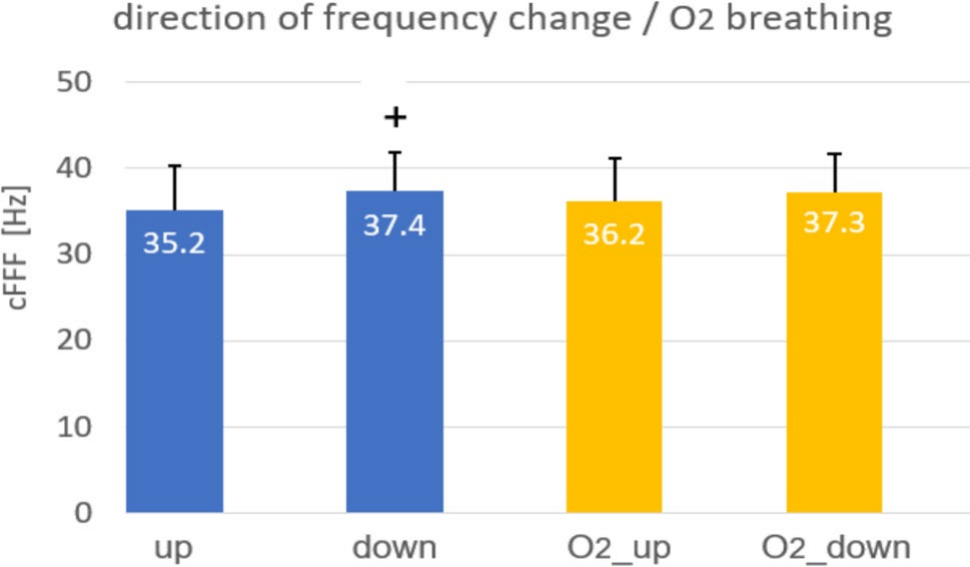
cFFFs depending on the direction of changing the frequency (left). The same dependency after 5-min after breathing normobaric O_2_ (right). ^+^*p* < 0.05 vs from down

Experiment 3*.* Possible effects of O_2_ breathing on cFFFs were investigated in a subgroup of 28 divers. The control measurements showed that a continuous decrease in FF led to significantly higher cFFF values compared to an increase in FF (35.2 vs. 37.4 Hz, *p* < 0.01) (Fig. 4; left).

After 5-min normoxic O_2_ breathing, the cFFF values remained nearly unchanged from the control. Again, the cFFF values were higher with a continuous decrease in FF than with an increase (36.2 vs. 37.3 Hz; n.s.) (Fig. 4, right).

**Fig. 5:**
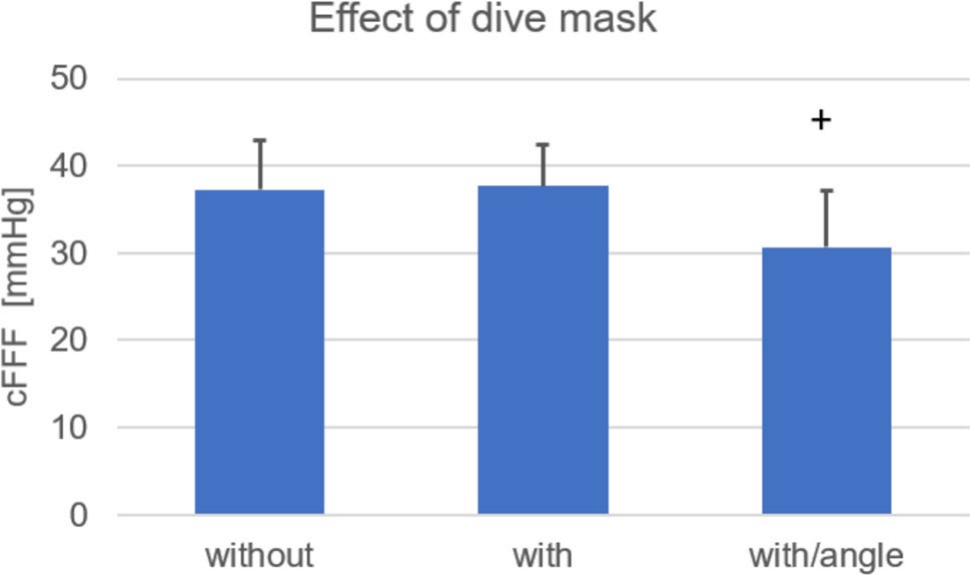
TcFFFs without a mask, with a mask and with a mask and diagonal incidence of the light beam on the mask lenses. ^+^*p* < 0.01 vs without and vs with mask

Experiment 4*.* In a subgroup of 24 divers, cFFF values were assessed at the pool-side for three conditions: no mask, with mask, and with mask but with an angle of 30° between the flicker light beam and the retinal plane. Wearing a mask had no effect on the cFFF values, but the diagonal incidence of the light beam on the mask lenses and the retinal plane led to a significant cFFF decrease compared to values without and with mask (37.3 vs 37.7 vs 30.7 Hz, respectively, *p* < 0.01 Fig. 5).

## Discussion

Our four experiments were designed to address potential confounders in critical flicker fusion frequency (cFFF) measurements, providing caveats to minimize the risk of misinterpretation. To ensure consistency and control, nearly 500 cFFF values were measured by the same individual using the same flicker device, with consistent geometry, lighting, and ambient temperature conditions maintained throughout.

The main findings are. Experiment 1 dealt with the demographic characteristics of the 174 participants. While no dependencies on gender or BMI were observed, cFFF values significantly decreased in participants over the age of 60, suggesting the potential need for age-specific standards. Despite stringent controls, the cFFF values among our 174 participants exhibited a 13% standard deviation.

Even repeated measurements in Experiment 2 on the same individual showed a 5% deviation. This variability suggests that differences between control and intervention groups in related studies must exceed these thresholds to be considered significant.

The same experiment explored the debated effects of lighting conditions. Over an 85-days period, exposure to moderately varying lighting environments (artificial, cloudy, or sunlight) did not significantly impact cFFF values.

In Experiment 3, we demonstrate that the direction of frequency change significantly influences cFFF: decreasing the frequency yields higher cFFF values compared to increasing the frequency.

Within this experiment, we also investigated the effects of normobaric oxygen on cFFF. Our results showed that normobaric oxygen had no impact on cFFF.

Experiment 4 focused on geometric alignment, highlighting the critical importance of maintaining orthogonal alignment between the light source and the fovea. Achieving correct alignment, which is particularly challenging in field studies like diving, is essential for obtaining accurate cFFF measurements, as misalignment can result in submaximal values.

Historically, cFFF has been a common screening tool for eye diseases (Barton and Rizzo 1994) and useful in diagnosing various neurological and internal conditions (Angeli et al. 2016). It has also been linked to early detection of Alzheimer’s disease (Curran and Wattis 2000) and hepatic encephalopathy (Kircheis et al. 2002) (Sharma 2017).

With extensive databases of healthy patients from clinical studies, identifying disease-related cFFF changes is relatively easy. However, clinical diagnoses are sensibly not based solely on cFFF changes.

In the context of studies on inert gas narcosis (IGN) under increased pressures, cFFF measurements have provided valuable results over the last decade (Balestra et al. 2012) (Hemelryck et al. 2013) (Rocco et al. 2019) (Lafère et al. 2019) (Piispanen et al. 2021). However, one study challenges this view: in a dry chamber experiment, cFFF values remained unchanged when comparing 6 bar to 1 bar of air (Vrijdag et al. 2020). Based on their results, CFFF did not seem to be a sensitive tool for measuring gas narcosis in divers in our laboratory setting.

The first part of Experiment 1 was run to understand any role of a demographic background. Gender has already been found not to significantly affect mean cFFF values between male and female adults (Bernardi et al. 2007), a result that was confirmed by our study. However, if the cohort was as large as to comprise 1.000 participants, then a moderate difference was found, with males having 6% higher cFFF values than females (Kaur et al. 2020).

Additionally, our data show that cFFF values in individuals aged 20 to 50 decline only gradually, whereas those in participants over 60 are significantly lower compared to younger age groups, demonstrating a 'large effect' according to Cohen's criteria (Cohen 1988). These findings align well with previous research reporting a steady decline beyond 60 years (Pitts 1982), likely due to age-related reductions in prefrontal cortex function (Mewborn et al. 2015).

Aging is also associated with an increased prevalence of cataracts, which can reach up to 50%, depending on geographic factors (Hirvelä et al. 1995). However, the literature suggests that cataracts do not affect cFFF (Romo et al. 2005) (Douthwaite et al. 2007). Therefore, our findings indicate that the observed decline in cFFF is primarily age-related.

These findings, on the other hand, are important because, according to the ‘Law of Initial Value’ (Wilder 1962), cFFF changes due to interventions tend to be smaller in older individuals than in younger ones. As a result, effects in an older group may be attenuated and fail to reach statistical significance, whereas the same intervention in a younger group might yield significant results.

Our finding that standard deviation was significantly greater among older participants than among younger ones has two implications. First, it suggests that detecting statistically significant effects of interventions in older individuals is more challenging due to increased variation in cFFF values. Second, it indicates that this measure of the visual system's temporal resolution deteriorates at different rates among older people.

Since heavier animals have been shown to have lower cFFF values than lighter ones (Healy et al. 2013) we hypothesized that heavier humans might also exhibit reduced agility compared to lighter individuals. Therefore, in addition to analyzing the effects of gender and age, we investigated whether cFFF correlates with body weight. However, no such relationship was found in our cohort.

In the second part of Experiment 1 (*n *= 174), experienced divers exhibited a cFFF standard deviation of approximately 13% during the control phase under identical conditions. We, therefore, propose that intervention-related changes in cFFF should exceed one standard deviation; for example, at a cFFF of 40 Hz, a meaningful change would be ± 5 Hz. Smaller changes may be inconclusive, as factors such as the presence of multiple participants in the hyperbaric chamber or elevated temperatures at 6 bar could act as confounders, potentially increasing cFFF values compared to control (Lockhart 1971) (Jensen et al. 2017). Somewhat of surprise, one study found that cFFF had improved by 2.5% after pressure changes in a hyperbaric chamber (Tikkinen et al. 2013). Assuming a standard cFFF value of 40 Hz, the measured difference of approximately 1 Hz is statistically significant but lacks physiological relevance.

Experiment 2 aimed to assess intraindividual variations in cFFF in a single adult male over a 85-days period. Given that cFFF values reportedly decrease throughout the day, indicating a reduction in sensitivity (Walsh and Misiak 1966), our measurements were consistently taken in the morning under stable lighting conditions to avoid confounding factors. The results showed a cFFF threshold of 43.4 ± 2.1 Hz. It is emphasized that this variation, amounting to roughly 5%, occurred even though the observer and the participant were the same person, which means the result will have a bias. Thus, studies should aim to induce changes greater than 5% to account for small cohort sizes (e.g., around 25 participants or fewer).

Due to conflicting results in the literature, this experiment also aimed to evaluate the effect of different lighting conditions. An earlier study suggested that intense sunlight is probably not a significant factor (Łuczak and Sobolewski 2000). However, more recent findings contradicted this view, showing substantially higher cFFF values under bright daytime illumination compared to dusk (Rider et al. 2022).

During the 85 days, lighting conditions varied only moderately between artificial light, cloudy daylight, and sunshine. These findings suggest that cFFF assessments remain consistent as long as lighting conditions do not vary significantly.

Experiment 3 addresses the inconsistent results from studies on the O_2_ effects on cFFF. Vrijdag et al. (2020) reported that breathing O_2_ at 1 bar and 2.8 bar caused a significant decrease in cFFF. In contrast, Kot et al. (2015) found that the O_2_ -effects on neuronal excitability in young, healthy men were dose-dependent: 0.7 bar O_2_ did not affect cFFF, 1.4 bar O_2_ significantly decreased cFFF, while 2.8 bar O_2_ allowed for recovery of cFFF. These findings are concerning, especially since normobaric oxygen is widely used in clinical settings (Asfar et al. 2015), including for mild depression (Bloch et al. 2021), and a partial pressure of 1.4 bar is considered a safe upper limit for divers (Fock and Millar 2008).

Some of these discrepancies may be attributable to uncontrolled variables in previous research—such as variations in lighting conditions, ambient temperature, timing of measurements, or potential learning effects—all of which were carefully addressed and controlled for in our study.

In our experiment, 5-min breathing normobaric oxygen did not decrease cFFF, which is reassuring. However, it also did not increase cFFF, contrary to the hypothesis that increased O_2_ availability could improve neural activity by accelerating nerve conduction (Brerro-Saby et al. 2010).

In line, positive effects of short-term normobaric oxygen on cognitive abilities, such as memory, visuospatial, and verbal skills, have been noted relatively early (Moss et al. 1998). Functional MRI studies further support the idea that hyperoxia has a pronounced effect on brain neural activity (Sheng et al. 2017).

Consistently, 10-min breathing normobaric oxygen resulted in a pronounced 17% increase in cFFF, which suggests improved cognitive performance. This is supported by simultaneous tests from a recognized test battery (PEBL) that also showed positive results (Hemelryck et al. 2013). Similar findings were reported by Lafère et al. (2019), where increased environmental pressure (4 bar) and breathing nitrox were associated with higher cFFF values and greater brain activity compared to breathing air.

The partially counterintuitive results regarding O_2_ effects could likely be attributed to different environmental conditions, varying absolute and partial O_2_ pressures, duration of exposure, and the modulating effects of additional gases such as nitrogen or helium in the breathing mixture. It is likely that changes in cFFF are not solely due to one single gas but rather a combination of these factors.

The second part of this experiment addresses the direction of frequency change during cFFF measurements. In many cFFF studies, measurements typically begin at low flicker frequencies and gradually increase. When flicker frequencies were decreased, cFFF values were slightly higher by about 2% (Vrijdag et al. 2020). Other studies have also reported slightly higher cFFF values when frequencies are decreased, with variations of approximately 4% among cyclists (Clemente-Suárez and Diaz-Manzano 2019) and participants in a yoga group (Vani et al. 1996). Similarly, our investigation found cFFF values to be about 6% higher when frequencies were decreased. These methodological differences can lead to variations comparable to those reported in the literature as significant results of interventions.

The impact of frequency change direction aligns with the ‘Concept of Perceptual Constancy’ (Augustyn 1991), where an impression conforms more to the assumed object than the actual stimulus, such as flickering or steady light. Thus, values found with the decreasing approach seem to be more 'subjective,' suggesting that employing the increasing cFFF approach is more reliable.

Another methodological aspect worth mentioning is the learning effect, although we present no original data on this. Bernardi et al. (2007) found that cFFF values in the second test run increased significantly over the first by roughly 3%. Thus, if the first test run is performed as a control and the second after an intervention, differences may be introduced by the learning effect (Bernardi et al. 2007).

Experiment 4 focuses on the relationship between cFFF and retinal location, presenting an important consideration. The cFFF is primarily determined by the density distribution of receptor cells (cones and rods) on the retina (Fukuda 1979). When a light source flickers, the flicker is more pronounced when its image falls on the fovea compared to when viewed eccentrically. As a result, the highest cFFFs are found when light hits the fovea directly (Creed and Ruch 1932).

Wearing a dive mask yielded no differences in cFFF values, despite the different density of the glass between the flicker light source and the fovea. However, when the flicker light no longer fell perpendicularly on the plane of the mask lenses, the cFFF was reduced by 16% due to the flicker light entering the eye eccentrically (Creed and Ruch 1932).

Therefore, it is important to maintain both the distance between the light source and the observer’s eyes as well as the visual angle throughout the experimental protocol. This context is crucial as results may be affected by differences in the positions of the light source and the observer’s eyes.

For example, during scuba diving, measurements are taken on the surface as well as at varying depths, where it is difficult to consistently maintain stable positions.

Additionally, movement of the light source or the observer can also affect cFFF values. An extreme example of potential error is eye motion, particularly due to saccades, which can significantly affect cFFF measurements. During saccades, humans can perceive flicker frequencies as high as 2 kHz (Roberts and Wilkins 2013).

### Limitations

The aim of our four experiments was to highlight conditions that may distort the accurate determination of the cFFF threshold. It is, of course, true that other confounders exist, as previously described (Balestra et al. 2018). In this original work, confounders such as varying illumination intensities, changes in observer, and the presence of ocular pathologies were identified. An unusual confounder—microgravity—was also investigated during parabolic flights, where cFFF thresholds were found to be significantly increased. This finding is particularly relevant considering the large number of studies conducted in divers.

The age-related decline in cFFF has long been well documented in groups of ophthalmologically healthy individuals. We confirm this association in a cohort of experienced recreational divers. However, our sample size was not sufficient to determine whether potential diving-related differences exist in the rate of age-dependent cFFF decline.

In some studies, the cFFF threshold is determined by repeated measurements within each participant, allowing for more reliable threshold estimation and improved detection of outliers. This approach likely yields thresholds with reduced variability, increasing the likelihood that intervention effects reach statistical significance. In contrast, single or duplicate measurements may act as confounders, particularly when conducted by inexperienced observers or with poorly instructed participants.

Another study recommends expressing the effects of interventions on cFFF as relative changes, i.e., percentage values (Balestra et al. 2018). The advantage of this approach is that it helps control for variations introduced by different flicker systems, making results from different research groups more comparable. However, in our four experiments, the primary goal was not to demonstrate intervention effects but to identify factors that could confound the measured cFFF values.

A fourth consideration is that administering oxygen for only 5 min may be insufficient to elicit measurable changes in cFFF. However, at least two published studies support the use of brief oxygen exposures: Scholey et al. (1999) observed a temporary improvement in reaction time during cognitive testing in young adults following oxygen administration, although the effect was short-lived and not significant for more complex tasks (Scholey et al. 1999). Similarly, Moss et al. (1998) reported slight improvements in word recognition tasks after short-term 100% O₂ inhalation under normobaric conditions (Moss et al. 1998). The transient nature of these effects does not undermine their relevance for the present study, as cFFF measurements were conducted immediately after oxygen administration, within the expected window of effect.

## Conclusion

cFFF is a well-established metric for assessing fatigue, mental workload, and the effects of inert gas narcosis (IGN). However, results can sometimes be unintuitive or contradictory, owing to variations in breathing gases, environmental pressures, measurement timing, and other potential confounding factors. In our study, we confirmed age-related differences in cFFF and demonstrated that the direction of frequency change (ascending vs. descending) significantly affects results. We also underscore the importance of controlling for major differences in illumination and temperature between control and post-intervention phases. Finally, maintaining consistent geometry between the light source and the retina is essential for reliable measurements.

Further research employing cFFF and cognitive competence tests should aim to further clarify the effects of IGN and O_2_-induced cerebral arousal, considering environmental pressures and exposure durations. Success in this area requires the avoidance of confounders and careful attention to potential caveats.

## Abbreviations

CCRs: Closed-circuit rebreathers (CCRs)
cFFF: Critical flicker fusion frequency
Nx32: Nitrox32
SCUBA: Self-contained underwater breathing apparatus
